# Association of Biomarkers of Systemic Inflammation with Organic Components and Source Tracers in Quasi-Ultrafine Particles

**DOI:** 10.1289/ehp.0901407

**Published:** 2010-02-02

**Authors:** Ralph J. Delfino, Norbert Staimer, Thomas Tjoa, Mohammad Arhami, Andrea Polidori, Daniel L. Gillen, Michael T. Kleinman, James J. Schauer, Constantinos Sioutas

**Affiliations:** 1 Department of Epidemiology, School of Medicine, University of California–Irvine, Irvine, California, USA; 2 Department of Civil and Environmental Engineering, Viterbi School of Engineering, University of Southern California, Los Angeles, California, USA; 3 Department of Civil Engineering, Sharif University of Technology, Tehran, Iran; 4 Department of Statistics, School of Information and Computer Sciences, University of California–Irvine, Irvine, California, USA; 5 Occupational and Environmental Medicine Division, Department of Medicine, School of Medicine, University of California–Irvine, Irvine, California, USA; 6 Environmental Chemistry and Technology Program, University of Wisconsin–Madison, Madison, Wisconsin, USA

**Keywords:** air toxics, biomarkers of effect, cytokines, epidemiology, longitudinal data analysis

## Abstract

**Background:**

Evidence is needed regarding the air pollutant components and their sources responsible for associations between particle mass concentrations and human cardiovascular outcomes. We previously found associations between circulating biomarkers of inflammation and mass concentrations of quasi-ultrafine particles ≤ 0.25 μm in aerodynamic diameter (PM_0.25_) in a panel cohort study of 60 elderly subjects with coronary artery disease living in the Los Angeles Basin.

**Objectives:**

We reassessed biomarker associations with PM_0.25_ using new particle composition data.

**Methods:**

Weekly biomarkers of inflammation were plasma interleukin-6 (IL-6) and soluble tumor necrosis factor-α receptor II (sTNF-RII) (*n* = 578). Exposures included indoor and outdoor community organic PM_0.25_ constituents [polycyclic aromatic hydrocarbons (PAHs), hopanes, *n*-alkanes, organic acids, water-soluble organic carbon, and transition metals]. We analyzed the relation between biomarkers and exposures with mixed-effects models adjusted for potential confounders.

**Results:**

Indoor and outdoor PAHs (low-, medium-, and high-molecular-weight PAHs), followed by hopanes (vehicle emissions tracer), were positively associated with biomarkers, but other organic components and transition metals were not. sTNF-RII increased by 135 pg/mL [95% confidence interval (CI), 45–225 pg/mL], and IL-6 increased by 0.27 pg/mL (95% CI, 0.10–0.44 pg/mL) per interquartile range increase of 0.56 ng/m^3^ outdoor total PAHs. Two-pollutant models of PM_0.25_ with PAHs showed that nominal associations of IL-6 and sTNF-RII with PM_0.25_ mass were completely confounded by PAHs. Vehicular emission sources estimated from chemical mass balance models were strongly correlated with PAHs (*R* = 0.71).

**Conclusions:**

Traffic emission sources of organic chemicals represented by PAHs are associated with increased systemic inflammation and explain associations with quasi-ultrafine particle mass.

Cardiovascular hospital admissions and mortality have been associated with ambient mass concentrations of fine particulate matter (PM) air pollution ≤ 2.5 μm in aerodynamic diameter (PM_2.5_) (Pope and Dockery 2006). Questions remain regarding the underlying causal chemical components and sources responsible for these associations. A recent time-series study of 106 U.S. counties showed stronger associations of cardiovascular hospital admissions with countywide averages of PM_2.5_ when there were higher fractions of elemental carbon (EC), nickel (Ni), and vanadium (V), suggesting that important sources included fossil fuel combustion, biomass burning, and oil combustion (Bell et al. 2009).

Unlike PM_2.5_, ultrafine particles (UFPs; generally defined as < 0.1 μm in diameter) are not regulated by the U.S. Environmental Protection Agency (EPA), yet this is the size fraction that may have the highest toxic potential because it has magnitudes greater number concentrations and surface area than the larger particles that dominate PM_2.5_ mass (Oberdörster et al. 2005). On that large surface area, UFPs carry and deliver redox-active organic chemicals, including polycyclic aromatic hydrocarbons (PAHs), to the respiratory tract in disproportionately higher concentrations than do larger particles (Ntziachristos et al. 2007), possibly leading to a cascade of effects related to oxidative stress and inflammation in the lungs and at extrapulmonary sites (Delfino et al. 2005). These and other effects could underlie associations of morbidity and mortality with air pollutants.

Except for some studies with personal or microenvironmental air pollution data (Chan et al. 2004; Delfino et al. 2008, 2009; Folino et al. 2009; Vinzents et al. 2005), regional ambient air monitoring has been the primary data source used in epidemiologic research on the importance of UFP exposure to cardiovascular outcomes and circulating biomarkers in individual-level studies (de Hartog et al. 2003; Henneberger et al. 2005; Ibald-Mulli et al. 2004; Lanki et al. 2008; Pekkanen et al. 2002; Rückerl et al. 2006, 2007; Timonen et al. 2006). These studies of ambient air were all conducted in Europe, and UFPs were measured as particle number concentrations at central regional sites. Exposure error from the use of ambient data is likely, because air monitors may be far from subject locations and subjects may be exposed to pollutants from local sources, including traffic. UFPs have much higher spatial variability than does PM_2.5_ (Sioutas et al. 2005), so exposure error is likely. In addition, UFP mass and particle number do not specifically indicate which particle components or sources are important, although generally in urban areas UFP compositions are dominated by organic chemicals and EC and originate from combustion sources.

We conducted a panel cohort study of elderly subjects with a history of coronary artery disease living in the Los Angeles Basin. This is considered a population that may have among the greatest susceptibility to the adverse effects of air pollution (von Klot et al. 2005). We made repeated measurements of blood biomarkers and air pollutant exposures. To assess the potential importance of UFPs to cardiovascular health, we measured quasi-ultrafine particle mass < 0.25 μm in diameter (PM_0.25_). To address the issue of exposure error, we monitored PM_0.25_ at the retirement communities of subjects. We previously reported positive associations of blood biomarkers of inflammation with PM_0.25_ but not with larger accumulation-mode particles 0.25–2.5 μm in diameter (PM_0.25–2.5_) (Delfino et al. 2009). However, particle mass alone does not provide sufficient information about composition or sources. We also previously found positive associations between the biomarkers and PM_2.5_ EC (Delfino et al. 2009). Based on these findings, we hypothesized that traffic emission sources of organic chemicals in PM_0.25_ would be positively associated with systemic inflammation. In the present analysis, we aimed to better delineate which underlying PM components may be responsible for the associations we observed for EC and PM_0.25_ mass using new data on chemical species in the archived PM_0.25_ filter samples.

## Materials and Methods

### Population and design

This was a longitudinal study of repeated measures where each subject acted as his or her own control over time. This limits the impact of confounding by between-subject characteristics. We recruited subjects from four retirement communities. Subjects were eligible for participation if they had a confirmed coronary artery disease history and were ≤ 65 years of age, nonsmokers, and unexposed to environmental tobacco smoke. Of 105 volunteers, 21 were not eligible, 19 dropped out, 2 had too few blood draws or valid biomarker data (> 5 of 12 weeks), and 3 had insufficient biomarker data due to exclusions for frequent infections, leaving 60 subjects. We excluded biomarker measurements during weeks with acute infectious illnesses given their well-known impact on measured biomarkers. Table 1 lists subject characteristics.

Two retirement communities were studied in 2005–2006 and two in 2006–2007. Subjects were followed for a total of 12 weeks with weekly blood draws for circulating biomarkers of inflammation in plasma. Each subject contributed 5–12 weekly blood draws (*n* = 578 total samples).

Each community was studied in two 6-week seasonal phases, a warmer period characterized by higher photochemistry followed by a cooler period characterized by higher air stagnation and lower mixing heights. This seasonal approach was intended to increase the variability in pollutant characteristics, with higher secondary organic aerosols (SOAs) in the warmer phase and higher primary organic aerosols (POAs) in the cooler phase when traffic-related air pollutants increase at ground level. POAs are formed during or shortly after the combustion of fossil fuels. SOAs are largely photochemically produced from gas-to-particle conversion when volatile reactive organic gases from anthropogenic and biogenic sources, and anthropogenic semivolatile organic compounds (SVOCs), are oxidized to form low-volatility products that condense to produce SOAs. There are few data on the importance of variations in this multipollutant characteristic of PM to human health outcomes. In the present study, POAs are represented by PAHs and hopanes, whereas SOAs are represented by water-soluble organic carbon (WSOC) and organic acids. Most PAHs are considered to be components of POAs. Hopanes are found in the lubricant oils of diesel and gasoline vehicles and are thus tracers of primary vehicular aerosols in the Los Angeles Basin (Schauer et al. 1996, 2000). WSOC (Snyder et al. 2009) and organic acids (Robinson et al. 2006) are tracers of SOAs, although a fraction of WSOC comes from biomass burning (Docherty et al. 2008).

The research protocol was approved by the Institutional Review Board of the University of California–Irvine, and we obtained informed written consent from subjects.

### Biomarkers

We focused on an informative set of biomarkers of inflammation from the previous analysis of peripheral blood biomarkers and PM_0.25_ mass (Delfino et al. 2009). We drew blood samples in ethylenediaminetetraacetic acid tubes on Friday afternoons and processed them and froze the plasma on site within 30 min. Samples were stored at −80°C until assayed. Plasma biomarkers were thawed and assayed using 96-well immunoassay kits for the proinflammatory cytokine interleukin-6 (IL-6) and the cytokine receptor–soluble tumor necrosis factor-α (TNF-α) receptor II (sTNF-RII; Quantikine HS, R&D Systems, Minneapolis, MN). sTNF-RII has a longer half-life than TNF-α (Aderka 1996) and may thus better reflect sustained or lagged effects. Thawed erythrocyte lysates were assayed spectrophotometrically for activities of the antioxidant enzymes copper/zinc-superoxide dismutase (Cu,Zn-SOD) and glutathione peroxidase-1 (GPx-1) (Cayman Chemical, Ann Arbor, MI, USA). Cu,Zn-SOD and GPx-1 values were normalized to units per gram of hemoglobin. These and related biomarkers are predictive of cardiovascular disease risk (Flores-Mateo et al. 2009; Kritchevsky et al. 2005; Pai et al. 2004).

### Exposures

The methods used to measure components and their relevance to sources of PM_0.25_ are described in detail in the Supplemental Material [Chemical Measurement Methods (doi:10.1289/ehp.0901407)] and by Arhami et al. (2010). There we also discuss in detail differences by season and community and describe the relation between indoor and outdoor measurements.

Air sampling occurred in the immediate outdoor environment of each retirement community and at an indoor site located in the common areas of the main community buildings. The indoor data are thus representative to some degree of the same indoor environment of each subject. Our main interest here is in the effects of outdoor-source PM components.

More than 5 days before each blood draw, we collected indoor and outdoor size-segregated particle samples using Sioutas Personal Cascade Impactors (SKC Inc., Eighty Four, PA, USA) with Zefluor filters (3-μm pore size; Pall Life Sciences, Ann Arbor, MI, USA). We evaluated components only in the quasi-ultrafine fraction (PM_0.25_). Mass concentrations were determined gravimetrically by weighing the impactor filters and substrates with a microbalance (uncertainty, ± 2 μg; Mettler-Toledo, Columbus, OH, USA) in a temperature-controlled and relative humidity–controlled room.

The five weekly PM_0.25_ filters were composited for chemical analyses. These composites were cut into three sections (one half-section and two quarter-sections). We analyzed the composited half section for 92 different organic compounds using gas chromatography/mass spectrometry (GC/MS) (Stone et al. 2008). For the present analysis, compounds are grouped by their structures, which is the primary control of their chemical interactions. Selected representative organic components were grouped as PAHs, organic (*n*-alkanoic) acids, *n*-alkanes, and hopanes [see Supplemental Material, Table 1 (doi:10.1289/ehp.0901407)]. PAHs were further subdivided into low- (two- to three-ring), medium- (four-ring), and high- (five-ring or larger) molecular-weight PAHs (LMW, MMW, and HMW, respectively), which is loosely connected to volatility and solubility.

The first composited quarter-section was digested with concentrated acid using microwave digestion followed by analysis to determine 52 trace elements using high-resolution inductively coupled plasma mass spectrometry (Finnigan Element 2; Thermo Fisher Scientific, Waltham, MA, USA) (Herner et al. 2006). We focused our analyses of exposure–response relationships on key transition metals that can generate reactive oxygen species by Fenton-type reactions: vanadium (V), chromium (Cr), iron (Fe), nickel (Ni), copper (Cu), manganese (Mn), lead (Pb), and zinc (Zn).

The second composited quarter was analyzed for WSOC using a General Electric Sievers Total Organic Carbon Analyzer (GE Analytical Instruments, Boulder, CO, USA).

The remaining composited half was analyzed for organic tracer compounds by GC/MS along with field blanks, laboratory blanks, spiked samples, and standard reference material (Urban Dust Standard Resource Material 1649a; National Institute of Standards and Technology, Gaithersburg, MD, USA). Spike recovery after correction for internal standard recoveries was in the range of 96–110% for PAHs, 99–104% for hopanes, and 68–136% for *n*-alkanes. Blank concentrations of MMW PAHs, HMW PAHs, and hopanes were below analytical detection limits (~ 10 pg/m^3^ air). The method detection limits for remaining compounds were limited by field and laboratory blanks. Uncertainties for each measurement were estimated based on analytical uncertainties and uncertainties from the blank correction and were used to determine if each measurement was statistically different from zero. The precision of the spike and standard reference material analyses was used to estimate method precision (> 20% for all PAHs, hopanes, and *n*-alkanes).

### Statistical analysis

We analyzed relations of repeated (within-subject) measures of biomarkers to air pollutant exposures with linear mixed effects models. Random effects were estimated at the subject level, nested within seasonal phase and community, to account for correlated within-individual repeated measures. To focus estimates of associations at the subject level, we adjusted for between-community and between-phase exposure effects as proposed by Janes et al. (2008) by using exposures that were mean-centered across community and phase [see Supplemental Material, Regression Model, Mean Centering Method (doi:10.1289/ehp.0901407)]. We decided *a priori* to adjust for 5-day average temperature. Magnitudes of association from the mixed models are expressed at pollutant interquartile ranges (IQRs; 25th–75th percentile) to allow strengths of association for different pollutants to be compared by limiting differences due to units of measurement or concentration range.

We evaluated the covariance structure using empirical variograms and found models were best fit as an autoregressive-1 correlation structure. We performed residual analyses to examine deviations from standard linear mixed model assumptions and the presence of influential observations. We found four influential high outliers for IL-6 > 10 pg/mL that were reset to 10 pg/mL (upper limit of its standard curve) to obtain more representative estimates of association. In a model for 5-day average PM_0.25_, including the outliers resulted in an association of 0.41 pg/mL [95% confidence interval (CI), 0.00–0.82] per interquartile change in PM_0.25_ of 7.37 μg/m^3^, whereas resetting them to 10 pg/mL resulted in an association of 0.26 pg/mL (95% CI, −0.06 to 0.57). It is important that in the previous analysis of PM_0.25_ mass (Delfino et al. 2009), the associations with 1-day and 3-day average PM_0.25_ were stronger and had narrower 95% CIs than did the 5-day average for both IL-6 and sTNF-RII.

In exploratory analyses, we retested models for erythrocyte antioxidant enzymes (Cu,Zn-SOD and GPx-1) from our previous publication (Delfino et al. 2009). Random slopes and individual autoregressive models showed small, highly influential subject clusters (seven subjects) with positive associations between air pollutants and antioxidant enzymes, whereas most of the remaining 53 subjects showed inverse associations. Details of these clusters and their interpretation are presented elsewhere (Delfino et al. 2009). We present these data-driven results with the new air pollutant exposure data primarily in the Supplemental Material, Table 3 (doi:10.1289/ehp.0901407).

## Results

Table 2 provides descriptive statistics for the measured exposures. Seasonal differences were greatest for MMW PAHs, HMW PAHs, and *n*-alkanes, which were higher in the cool season, and for WSOC, which was higher in the warm season, as expected because of photochemistry. Indoor/outdoor ratios were close to 1.0 for PAHs and hopanes, and indoor–outdoor correlations were strong (median *R* was 0.60 for PAH species and 0.74 for hopane species) (Arhami et al. 2010). This suggests high penetration of these outdoor PM_0.25_ components into indoor environments and that measured indoor components were largely of outdoor origin. On the other hand, indoor/outdoor ratios were high for *n*-alkanes and *n*-alkanoic acids, with generally low indoor/outdoor correlation coefficients (Arhami et al. 2010). This suggests that indoor sources influenced the indoor levels of *n*-alkanes and *n*-alkanoic acids.

Table 3 shows a correlation matrix for measured outdoor organic components. We found moderate to strong correlations between PM_0.25_ mass, PAHs, and hopanes. We also found small negative correlations of these species with organic acids and small positive correlations with WSOC, suggesting that POA and SOA concentrations are relatively independent of each other at the study sites.

To further improve our understanding of the clearly positive associations of biomarkers with summed PAH compounds presented below, we used the chemical mass balance model (CMB) source apportionment estimates from Arhami et al. (2010) to evaluate the possible sources of PAHs. We briefly summarize methods and source apportionment results in the Supplemental Material, Chemical mass balance (CMB) model (doi:10.1289/ehp.0901407). Table 4 shows a correlation matrix for the relation of PAHs to the CMB-estimated sources. Strong correlations are seen for total PAHs with vehicular emission sources, whereas the apportioned mass from other sources shows weak to null correlations.

**Figure 1:**
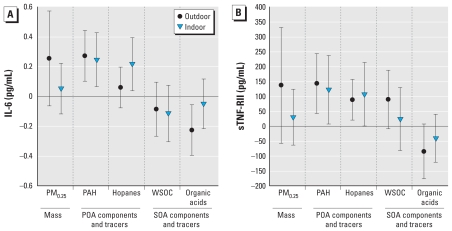
Associations of biomarkers with 5-day average outdoor and indoor community PM_0.25_ mass, and markers of POAs and SOAs. (*A*) IL-6. (*B*) sTNF-RII. Expected change in the biomarker (adjusted coefficient and 95% CI) corresponds to an IQR increase in the air pollutant concentration (see Table 2), adjusted for temperature.

In the mixed-model regression analyses, we found positive associations of circulating biomarkers of inflammation (IL-6 and sTNF-RII) with organic components (Table 5, Figure 1). We found the strongest associations with biomarkers for both indoor and outdoor PAHs, including LMW, MMW, and HMW PAHs. The next strongest associations were for hopanes. Indoor but not outdoor hopanes were associated with IL-6, whereas both indoor and outdoor hopanes were associated with sTNF-RII.

Outdoor WSOC (a marker of SOAs) was positively associated with sTNF-RII, but confidence limits crossed 1.0 (*p* < 0.14), and we found no other associations with SOA markers. The outdoor organic acids (another marker of SOAs) showed a pattern opposite to that of the POA markers, with largely negative regression coefficients in relation to biomarkers of inflammation. To assess whether this was due to inverse correlations with PAHs, we coregressed outdoor total PAHs with outdoor organic acids. We found that associations with PAHs and with organic acids decreased in magnitude to small degrees when coregressed, suggesting that the negative regression coefficients for organic acids with biomarkers of inflammation may be attributed to other unmeasured factors or chance.

**Figure 2:**
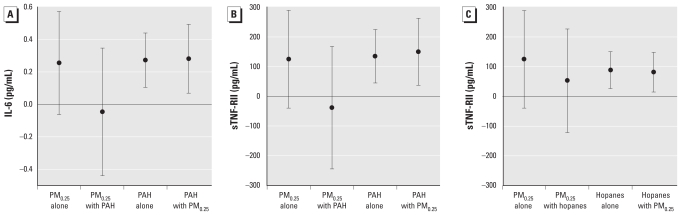
Associations of circulating biomarkers of inflammation with outdoor PM_0.25_ mass coregressed with outdoor total PAHs and hopanes in PM_0.25_. (*A*) IL-6, PAHs, and PM_0.25_. (*B*) sTNF-RII, PAHs, and PM_0.25_. (*C*) sTNF-RII, hopanes, and PM_0.25_. Expected change in the biomarker (adjusted coefficient and 95% CI) corresponds to an IQR increase in the air pollutant concentration (see Table 2), adjusted for temperature.

We then tested two-pollutant regression models that included both outdoor PM_0.25_ mass and total PAHs to assess whether PAHs explained the nominal association with mass. We found that IL-6 and sTNF-RII associations with mass were completely confounded by PAHs in that the regression coefficient for mass decreased to just below zero and the regression coefficient for PAHs was nearly unchanged (Figure 2A, B). We found a similar effect for hopanes, which confounded the nominal association of PM_0.25_ mass with sTNF-RII (Figure 2C). The variance inflation factor was < 3.5 for exposures, thus showing little evidence of multicollinearity.

Transition metals were not associated with the biomarkers [see Supplemental Material, Table 2 (doi:10.1289/ehp.0901407)].

As previously shown (Delfino et al. 2009), the analysis of the relation of erythrocyte antioxidant enzymes (Cu,Zn-SOD and GPx-1) to air pollutants among all 60 subjects showed regression coefficients were largely negative, suggesting inverse associations, but most upper confidence limits crossed 1.0 (see Supplemental Material, Table 3 (doi:10.1289/ehp.0901407)]. The exploratory analysis showed that among seven subjects previously identified as a “positive responder group” (Delfino et al. 2009), we found largely positive associations of Cu,Zn-SOD and GPx-1 with air pollutants, and lower confidence limits were > 1.0 for outdoor PM_0.25_ mass and several other exposures. In the 53 subjects previously identified as a “negative responder group,” we found inverse associations of Cu,Zn-SOD and GPx-1 with indoor and outdoor total, LMW, MMW, and HMW PAHs and with hopanes (all markers of exposures linked to primary combustion). Indoor WSOC was inversely (*p* < 0.07) associated with Cu,Zn-SOD, but we found no other associations with SOA markers in the negative responder group. Confidence limits were wider for GPx-1 than for Cu,Zn-SOD.

## Discussion

To our knowledge, this is the first report from a panel cohort study to show associations of circulating biomarkers of response in human subjects to specific PM organic compound classes. The measured chemicals serve as indicators and tracers for air pollutant sources and for classes of chemicals with the potential for redox activity in the body. Our prior work has focused on carbonaceous aerosols that provided some differentiation between POAs and SOAs by showing associations of biomarkers of inflammation with primary PM_2.5_ organic carbon (OC) but not secondary PM_2.5_ OC (a marker of SOAs) (Delfino et al. 2008, 2009). In the present analysis, we found the strongest biomarker associations with air pollutant variables for all molecular weight classes of PAHs and specific source markers of vehicular emissions (hopanes) measured in PM_0.25_ with GC/MS. Furthermore, two-pollutant models of the relation between the biomarkers of systemic inflammation and both total PAHs and PM_0.25_ mass showed that mass associations were completely explained by PAHs. Given the results of the chemical mass balance analysis [see Supplemental Material, Chemical mass balance (CMB) model (doi:10.1289/ehp.0901407) and Arhami et al. 2010], we infer that the confounding of nominal associations between biomarkers and PM_0.25_ mass by PAHs was through a common set of sources. PAHs likely serve here as a surrogate for redox-active PM chemical components as evidenced in experimental models (Riedl and Diaz-Sanchez 2005). For example, PAHs from diesel exhaust particles and oxidized derivatives of PAHs such as quinones lead to the generation of reactive oxygen species and subsequent oxidant injury and inflammatory responses, including the expression of nuclear transcription factor-κB (NFκB) (Riedl and Diaz-Sanchez 2005). NFκB increases the transcription of cytokines and acute-phase proteins that are predictive of coronary artery disease risk (Pai et al. 2004). PAHs can induce oxidative stress responses after biotransformation to quinones by cytochrome P450 1A1 (Bonvallot et al. 2001), perhaps after delivery from the lungs to systemic targets.

In the Los Angeles Basin, most outdoor PAHs in PM_0.25_ are expected to be from mobile sources (Schauer et al. 1996), and the CMB exposure correlations are consistent with this expectation. PAHs were also correlated with source markers of vehicular emissions (hopanes). Hopanes are the most unambiguous source marker of traffic emissions. However, the moderate but not strong correlation between hopanes and PAHs suggests that the measured PAHs include a different subset of mobile sources than that of hopanes. This may in part be due to the variability in PAHs relative to hopanes by combustion-related problems in the vehicle fleet (Lough et al. 2007).

Overall, the associations of biomarkers with PAHs and hopanes suggest that our previous findings of positive associations of biomarkers with PM_2.5_, EC, and primary OC (Delfino et al. 2009) were due to PM of mobile-source origin. PAHs are found in greater concentrations in the quasi-UFP range compared with larger particles (Ntziachristos et al. 2007), and this has been hypothesized to explain enhanced prooxidative and proinflammatory effects of urban UFPs in the lungs and peripheral target organs of rodents (Araujo et al. 2008). The increased biological potency of UFPs may be related to the content of organic chemicals that have the capacity to reduce oxygen, such as quinones and nitro-PAHs, for which PAHs may act, in part, as a surrogate (Ntziachristos et al. 2007) or as a source after biotransformation. From the present results we infer that, although PAHs may have an effect by themselves, they are also likely surrogates for other causal species we did not measure that are emitted from the same (traffic) sources.

We found little evidence that tracer variables for SOAs and related components (WSOC and organic acids) were associated with the circulating biomarkers in the expected direction. We have no explanation for the negative regression coefficients for organic acids with biomarkers. Although most of the SOAs are expected to be in larger PM > 0.25 μm, the present results are consistent with our finding of few biomarker associations with PM_2.5_ secondary OC or accumulation mode particle mass (PM_0.25–2.5_) in an earlier publication (Delfino et al. 2009). In that study, regression coefficients were also negative for IL-6 in some models with PM_0.25–2.5_ and with secondary OC. We speculate that components in outdoor SOAs estimated by our methods (e.g., organic acids), are mostly water soluble and highly oxygenated, and dissolve after deposition on the airway epithelium and then quickly react with extracellular macromolecules and cell membrane constituents. Thus, these PM components may not directly interact with the vasculature, although it has been hypothesized that inhaled particles lead to airway inflammatory responses and subsequent release of activated leukocytes and cytokines into the circulation (Mills et al. 2009).

An important limitation of our characterization of SOAs is that WSOCs and organic acids do not completely characterize the SOA fraction of PM, part of which may come from the photochemical oxidation of low-volatility vapors to form hydrophilic organic components, but whose chemical identity is largely unknown. These precursor vapors include SVOCs that are largely part of POAs. SVOCs evaporate from the particle phase during the process of atmospheric dilution and subsequently react with oxidant gases to form a significant fraction of SOAs (Robinson et al. 2007).

Lipid-soluble components of PM more closely associated with primary emissions, including PAHs, may become bioavailable after deposition followed by distribution of unmetabolized chemicals to the circulation and to extrapulmonary target sites (Gerde et al. 2001). It is also possible that a small fraction of toxic components is carried via various translocation mechanisms into the circulation on UFPs (Mühlfeld et al. 2008). However, translocation may account for a potentially insignificant amount of the impact of UFPs compared with the high retention of UFPs in the lungs (Möller et al. 2008), which may lead to sustained effects through the gradual transfer of redox-active components to the circulation over many days.

Although transition metals are known to be redox active, we found no consistent associations with the biomarkers measured, possibly because of low concentrations of these trace elements in the study areas.

Finding positive associations of biomarkers with both indoor and outdoor PAHs and hopanes along with the indoor/outdoor ratios of these organic components being close to 1.0 suggests that, even though people spend most of their time indoors, indoor air quality and PM exposures are strongly influenced by PM of outdoor origin. These findings are consistent with our previous analysis for the first half of this panel showing that CMB-estimated indoor PM of outdoor origin (particle number, EC, and primary OC) were associated with the biomarkers to a similar degree as outdoor PM (Delfino et al. 2008).

Briefly, the exploratory (data-driven) findings for GPx-1 and especially Cu,Zn-SOD are consistent with our previous findings for primary OC and EC (Delfino et al. 2009) and suggest antioxidant enzyme inactivation within erythrocytes by traffic-related pollutant components, including PAHs, among a subgroup of people. This inactivation is anticipated to increase oxidative stress and thus inflammation. This is potentially important because these enzymes likely represent important intermediate end points that have been linked to the risk of developing coronary artery disease in prospective cohort and other studies (Flores-Mateo et al. 2009). Given that these findings were far less clear when including the entire 60-subject panel (because a small subgroup of seven subjects had positive associations), these results should be viewed as hypothesis generating and retested in other populations. See Delfino et al. (2009) for further details and discussion concerning potential mechanisms of antioxidant enzyme inactivation versus up-regulation that may explain group differences.

Strengths of the present study lie in exposure measurements in each subject’s community microenvironment and in repeated biological marker assessments in a well-characterized patient sample. Limitations include the potential for unmeasured temporal confounding. However, we performed *a priori* adjustment for one of the largest sources of variability in inflammatory mediators that have been documented in the literature (infections), and we also accounted for temperature and for community and seasonal variability in exposures. We also acknowledge that the present study does perform multiple comparisons, although we did narrow the number of hypotheses being tested based on prior evidence of associations from the work of others and ourselves.

The results of the present study suggest that tracer components of mobile source emissions in PM_0.25_ are associated with increased systemic inflammation in a potentially susceptible population of elderly individuals. The measured biomarkers likely represent important intermediate end points (systemic inflammation) that have been linked to the risk of cardiovascular diseases in prospective cohort and other studies (Kritchevsky et al. 2005; Pai et al. 2004). The positive relation between air pollution and cytokine biomarkers may also be indicative of acute risk of adverse cardiovascular outcomes related to vascular dysfunction and atherothrombosis (Mills et al. 2009). We recently reported coherent associations between hourly ambulatory systolic and diastolic blood pressure and hourly air pollutant exposures in the present panel cohort, including stronger associations with primary PM_2.5_ OC compared with secondary PM_2.5_ OC (Delfino et al. 2010).

We conclude that U.S. EPA–regulated ambient PM_2.5_ mass measurements may not adequately represent risk to human health because they are uncharacterized by composition, source, or PM size distribution and are not necessarily representative of personal or local exposure. Confirmatory data are needed in other populations using measurements of organic components across several PM size fractions.

## Figures and Tables

**Table 1:** Subjects and biomarker outcomes.

Variable	Value
Age (years)	84.1 ± 5.60
Sex	
Male	34 (56.7)
Female	26 (43.3)
Cardiovascular history	
Confirmation of coronary artery disease^a^	
Myocardial infarction	27 (45.0)
Coronary artery bypass graft or angioplasty	20 (33.3)
Positive angiogram or stress test	10 (16.7)
Clinical diagnosis^b^	3 (5.0)
Congestive heart failure	13 (21.7)
Hypertension (by history)	42 (70.0)
Hypercholesterolemia (by history)	43 (71.7)
Medications	
Angiotensin-converting enzyme inhibitors and angiotensin II receptor antagonists	24 (40.0)
3-Hydroxy-3-methylglutaryl-coenzyme A reductase inhibitors (statins)	31 (51.7)
IL-6 (pg/mL)	2.42 ± 1.85
sTNF-RII (pg/mL)	3,610 ± 1,489

sTNF-RII, soluble tumor necrosis factor-α receptor II. Values are mean ± SD or *n* (%).

a Each category is hierarchical and excludes being in the above diagnostic category.

b Includes subjects with anginal symptoms relieved with nitrates plus echocardiogram and electrocardiographic evidence of past infarct.

**Table 2:** Descriptive statistics of outdoor measurements and indoor/outdoor (I/O) ratios of PM_0.25_ organic components and transition metals from 47 weeks of 5-day filter composites.

	Warm season				Cool season				
Exposure	Mean ± SD	IQR	Min/max	I/O ratio	Mean ± SD	IQR	Min/max	I/O ratio	IQR overall^a^
Organic components									
PM_0.25_ mass (μg/m^3^)	9.51 ± 3.46	7.24	4.67/14.7	0.88	8.65 ± 4.51	6.07	3.31/19.3	0.94	7.37
WSOC (μg/m^3^)^b^	0.52 ± 0.23	0.31	0.08/1.01	0.95	0.38 ± 0.23	0.39	0.06/0.94	0.94	0.37
PAHs (ng/m^3^)									
Total	0.88 ± 0.37	0.47	0.40/1.75	0.84	1.04 ± 0.61	0.73	0.40/2.70	0.99	0.56
LMW	0.38 ± 0.15	0.20	0.19/0.74	0.78	0.33 ± 0.15	0.19	0.17/0.73	1.02	0.19
MMW	0.26 ± 0.12	0.18	0.09/0.50	0.85	0.35 ± 0.24	0.33	0.09/0.96	0.74	0.24
HMW	0.24 ± 0.11	0.18	0.11/0.50	0.97	0.37 ± 0.24	0.32	0.14/1.01	1.04	0.21
Hopanes (ng/m^3^)	0.27 ± 0.34	0.36	0.06/1.57	1.00	0.25 ± 0.25	0.35	0.06/0.83	0.97	0.35
*n*-Alkanes (ng/m^3^)	36.3 ± 23.5	43.2	9.9/81.2	1.39	54.8 ± 111	15.9	11.7/500	1.30	29.4
Organic acids (μg/m^3^)	0.22 ± 0.17	0.30	0.06/0.54	5.05	0.26 ± 0.22	0.26	0.07/0.96	1.24	0.29

Transition metals (ng/m^3^)									
V	4.83 ± 2.07	2.10	1.66/11.3	0.75	2.10 ± 1.19	2.40	0.54/4.25	0.77	2.95
Cr	10.2 ± 30.2	2.21	0.00/139	0.89	0.26 ± 0.45	0.49	0.00/1.24	1.00	1.18
Mn	3.09 ± 2.88	3.10	0.00/13.8	0.57	2.02 ± 1.43	1.76	0.27/6.19	0.70	2.24
Fe	144 ± 127	167	0.00/588	0.49	92.5 ± 64.2	74.7	9.39/287	0.74	115
Ni	7.21 ± 18.0	3.51	0.00/82.8	0.83	0.20 ± 0.61	0.816	0.00/1.44	2.27	1.64
Cu	6.45 ± 4.35	5.50	0.35/16.0	0.64	4.69 ± 3.22	4.91	0.43/11.3	0.60	4.69
Zn	6.88 ± 4.16	6.39	0.00/15.8	0.78	6.08 ± 3.51	4.81	1.75/13.0	0.93	5.77

Abbreviations: max, maximum; Min, minimum.

a Overall IQR used in regression models to estimate expected change in the biomarker from exposure to the air pollutant.

b WSOC (μg C/m^3^) was multiplied by 1.8 to yield mass of organic components (μg/m^3^) according to Turpin and Lim (2001).

**Table 3:** Exposure correlation matrix for outdoor PM_0.25_ mass and organic components.

		PAH						
Pollutant	WSOC	Total	LMW	MMW	HMW	Hopanes	*n*-Alkanes	Organic acids
PM_0.25_ mass	0.25	0.45	0.44	0.38	0.39	0.31	0.17	−0.18
WSOC	1.00	0.39	0.41	0.29	0.40	0.31	0.15	0.09
PAHs								
Total		1.00	0.89	0.93	0.81	0.54	0.15	−0.19
LMW			1.00	0.79	0.66	0.63	0.24	−0.24
MMW				1.00	0.67	0.51	0.12	−0.33
HMW					1.00	0.41	0.20	−0.03
Hopanes						1.00	0.08	−0.26
*n*-Alkanes							1.00	−0.06

All exposures are mean centered by study community and seasonal phase, and results are Spearman rank correlations.

**Table 4:** Exposure correlation matrix for outdoor PAH and source apportioned mass.

PAH	Vehicular emissions	Biomass burning	Ship emissions	SOAs	RS dust	NSS sulfate	Sea salt	Unknown
Total	0.71	0.22	0.10	0.19	0.24	0.06	0.33	0.33
LMW	0.70	0.14	0.17	0.27	0.39	0.10	0.34	0.31
MMW	0.66	0.36	−0.01	0.04	0.19	−0.06	0.27	0.30
HMW	0.66	0.08	0.09	0.27	0.13	0.13	0.19	0.14

Abbreviations: RS, resuspended; NSS, non-sea salt. All exposures are mean centered by study community and seasonal phase, and results are Spearman rank correlations. Source apportioned mass data come from Arhami et al. (2010).

**Table 5:** Associations of biomarkers of systemic effect with indoor and outdoor 5-day average PM_0.25_ mass and organic components [regression coefficient (95% CI)].

Air pollutant	IL-6 (pg/mL)	sTNF-RII (pg/mL)
PM_0.25_ mass		
Indoor	0.05 (−0.12 to 0.22)	18 (−61 to 97)
Outdoor	0.26 (−0.06 to 0.57)	125 (−40 to 289)
WSOC		
Indoor	−0.11 (−0.30 to 0.08)	15 (−77 to 108)
Outdoor	−0.08 (−0.27 to 0.10)	63 (−19 to 145)
PAHs		
Total		
Indoor	0.25 (0.07 to 0.43)**	119 (16 to 223)*
Outdoor	0.27 (0.10 to 0.44)**	135 (45 to 225)**
LMW		
Indoor	0.30 (0.10 to 0.50)**	115 (−2 to 233)
Outdoor	0.22 (0.05 to 0.39)*	109 (19 to 200)*
MMW		
Indoor	0.28 (0.07 to 0.48)**	138 (22 to 254)*
Outdoor	0.30 (0.12 to 0.48)**	143 (47 to 238)**
HMW		
Indoor	0.18 (0.02 to 0.35)*	91 (1 to 181)*
Outdoor	0.26 (0.07 to 0.44)**	137 (39 to 234)**
Hopanes		
Indoor	0.22 (0.04 to 0.39)*	107 (10 to 204)*
Outdoor	0.06 (−0.08 to 0.20)	89 (26 to 151)**
*n*-Alkanes		
Indoor	0.01 (−0.03 to 0.06)	−6 (−27 to 16)
Outdoor	0.009 (−0.03 to 0.05)	14 (−6 to 34)
Organic acids		
Indoor	−0.05 (−0.22 to 0.12)	−36 (−109 to 37)
Outdoor	−0.22 (−0.39 to−0.06)**	−82 (−164 to 1)

Regression coefficients and 95% CIs are for the expected change in the biomarker among 60 subjects associated with an IQR change in the air pollutant (see Table 2), adjusted for temperature.

* *p* < 0.05

** *p* < 0.01.
